# Could hair cortisol in free-ranging cattle be a proxy of wolf predation patterns?

**DOI:** 10.1093/conphys/coag002

**Published:** 2026-01-28

**Authors:** Marta Rafael, Eliana Fonseca, Nuno Santos, Mónia Nakamura

**Affiliations:** Instituto de Ciências Biomédicas Abel Salazar (ICBAS), Universidade do Porto, 4050-313 Porto, Portugal; Instituto da Conservação da Natureza e das Florestas, I.P., 4704-538, Braga, Portugal; CIBIO/InBIO, Centro de Investigação em Biodiversidade e Recursos Genéticos, InBIO Laboratório Associado, Campus de Vairão, 4485-661 Vairão, Portugal; BIOPOLIS, Program in Genomics, Biodiversity and Land Planning, CIBIO, Campus de Vairão, 4485-661 Vairão, Portugal; CIBIO/InBIO, Centro de Investigação em Biodiversidade e Recursos Genéticos, InBIO Laboratório Associado, Campus de Vairão, 4485-661 Vairão, Portugal; BIOPOLIS, Program in Genomics, Biodiversity and Land Planning, CIBIO, Campus de Vairão, 4485-661 Vairão, Portugal

**Keywords:** *Bos taurus*, *Canis lupus*, chronic stress, enzyme-linked immunosorbent assay, glucocorticoids

## Abstract

Cortisol is a biomarker of grey wolf (*Canis lupus*) prey selection on wild ungulates. Throughout its range, wolves may prey on free-range livestock, leading to conflicts with humans. This can compromise wolf conservation through culling or poaching. We investigate whether glucocorticoid concentration could be a biomarker of individual prey selection by grey wolves that depredate on free-ranging cattle (*Bos taurus*). To achieve this, cortisol concentration in hair samples from live (*n* = 46) and wolf-preyed (*n* = 19) cattle was determined by enzyme-linked immunosorbent assays. The effects of intrinsic and extrinsic variables—namely age, sex and food availability—on hair cortisol concentration (HCC) were investigated through linear mixed models with farm as a random effect. The analysis revealed that, against our initial hypothesis, wolf-preyed cattle had significantly lower HCC than live cattle (*P* = 0.009). Additionally, HCC was lower in subadults than in adults (*P* = 0.002), and was negatively correlated with food availability in adults, but not in subadults (*P* = 0.003). These results suggest that predation risk does not necessarily equal long-term physiological stress. Alternatively, it may indicate that cattle chronically exposed to stressors (i.e. presenting higher HCC) may exhibit more effective anti-predatory behaviours. Additionally, food availability for cattle may influence wolf predation patterns, as cattle may expand their foraging area by exploring unfamiliar areas, thereby increasing the likelihood of predator encounters. Further research is required to understand the relationship between the multitude of stressors acting on free-range cattle and wolf prey selection, with the aim of assessing the risk of individual cattle and eventually managing predation risk and human–wolf conflict.

## Graphical Abstract

**Graphical Abstract:**
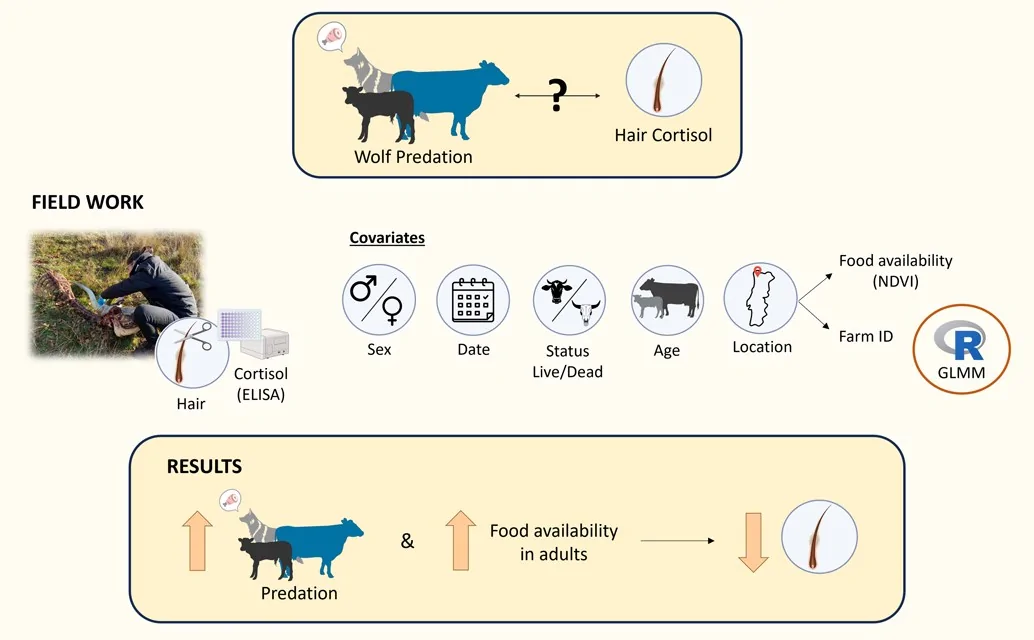

## Introduction

Animals are exposed to environmental stressors daily, eliciting a range of physiological and behavioural responses that enable them to survive and adapt to their surroundings (Herman *et al*., 2016; Romero and Wingfield, 2016). Exposure to a stressor activates the hypothalamic–pituitary–adrenal (HPA) axis, resulting in the release of glucocorticoids, specifically cortisol, in many mammal species. When exposed to an acute stressor, the increase in glucocorticoids is short-lived, returning to its previous state after the threat is over. In opposition, with chronic stressors, the coping mechanisms remain activated for extended periods, leading to increased cortisol concentration over longer periods (Romero and Wingfield, 2016).

Glucocorticoid concentrations in the organism can be determined through multiple techniques and sample matrices. The primary matrices used to measure glucocorticoids are blood, saliva, urine and faeces (Gormally and Romero, 2020). However, to evaluate the long-term HPA axis activity, keratinised tissues, such as hair, may present advantages over other matrices. Although the incorporation of glucocorticoids into hair is not entirely uncovered (Tallo-Parra *et al*., 2017; Colding-Jørgensen *et al*., 2023), the most accepted theory argues that glucocorticoids diffuse from follicular capillaries and integrate into the hair’s shaft during active growth (Russell *et al*., 2012). According to this theory, hair can be used to assess glucocorticoid concentration over longer periods (Gormally and Romero, 2020).

Frequent exposure to stressors can have lasting effects due to the prolonged release of glucocorticoids, jeopardising the animals’ well-being and negatively affecting their survival (Rakotoniaina *et al*., 2017). Animals’ health can become compromised, and comorbidities may arise, evidenced by immune suppression, infertility or decreased body condition (Sapolsky *et al*., 2000; Romero and Wingfield, 2016), and may increase their vulnerability to predation (Shave *et al*., 2019). In Canada, Shave *et al*. (2019) encountered higher hair cortisol concentration (HCC) in wolf-preyed bison (*Bison bison*) than those harvested by humans and a negative correlation between HCC and body condition. Hence, it was suggested that bison with higher HCC and lower body condition are more vulnerable to predation by wolves. Owing to this, understanding whether cortisol, as a chronic indicator of stress, is a suitable biomarker for wolf predation patterns in species other than bison can contribute to a better understanding of the ecology of predation by wolves.

The Iberian wolf population is comprised of > 300 packs inhabiting predominantly human-dominated landscapes (Chapron *et al*., 2014). During the 20th century, wolf distribution in the Iberian Peninsula declined, with wolves disappearing from most regions (Pimenta *et al*., 2023). In the Northwestern Iberian Peninsula, wolves prey mostly on domestic livestock due to low wild prey and high livestock densities, such as cows (*Bos taurus*), goats (*Capra hircus*), sheep (*Ovis aries*) or horses (*Equus caballus*) (Newsome *et al*., 2016). Such behaviour is facilitated by the often inadequate livestock husbandry or inefficient damage prevention measures, such as the absence or insufficient number of guarding dogs (Pimenta *et al*., 2018). The number of reported wolf attacks on livestock in Portugal has been increasing since 1996, with > 2500 attacks reported in 2014 (Álvares *et al*., 2015). Although livestock depredations are compensated in Portugal, they are not often adequately addressed and managed, leading to conflicts and retaliatory killing of wolves (Álvares *et al*., 2015; Pimenta *et al*., 2017). Simultaneously, the abundance of free-ranging dogs is increasing, with more attacks on livestock attributed to dogs (Lino *et al*., 2023). While in Portugal wolves have been strictly protected by law since 1988, the Spanish government has most recently changed the protection status of northern wolf populations, allowing legal hunting of northern wolf populations (BOE, Ley 1/2025, 2025). These management strategies may further jeopardise wolf conservation efforts in the Iberian Peninsula, modifying the ecological balance and prey dynamics (Licht *et al*., 2010; Frey *et al*., 2022).

This study aimed to assess HCC in free-ranging cattle in Northwestern Portugal, near the Spanish border, as a biomarker of the risk of predation by wolves. This objective was achieved by comparing the HCC of wolf-preyed and alive free-ranging livestock living within the territories of five wolf packs in Northwest Portugal (Nakamura *et al*., 2021), controlling for potential confounding effects of the variables age, sex and food availability. The working hypothesis was that predation and HCC had a positive correlation, with wolf-preyed cattle presenting higher HCC, as found for wild bison in Canada (Shave *et al*., 2019).

## Materials and Methods

### Study area

**Figure 1:**
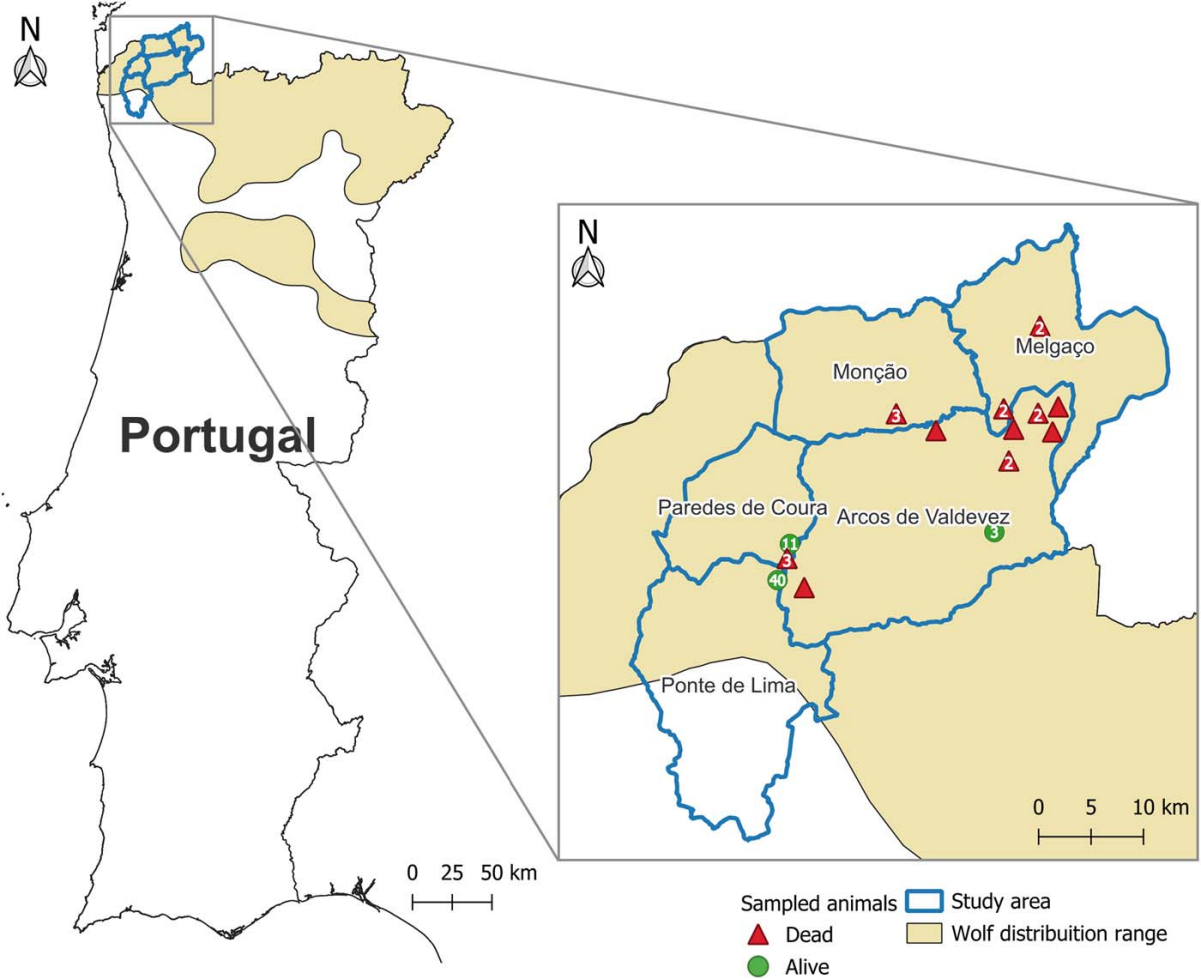
Map depicting the sampling geographical locations in the study area (northwest Portugal). Geo-reference locations of samples for wolf-killed sites (red triangles) and alive cattle (green dots). The number inside the triangles or dots represents the number of samples retrieved in each location. Map generated in QGIS version 3.34.3.

The study was conducted in a mountainous region of Northwest Portugal (Fig. 1), where low densities of wild ungulates, namely roe deer (*Capreolus capreolus*), red deer (*Cervus elaphus*), Iberian ibex (*Capra pyrenaica*) or wild boar (*Sus scrofa*) coexist with high cattle densities (10.2 cattle/km^2^—INE, 2021). Furthermore, cattle graze the mountains for most of the year with scarce or no vigilance from shepherds, known as extensive grazing system. In this region, most cattle are small-sized autochthonous breeds (mostly *Cachena*), with an average adult body weight between 250 and 290 kg (Brito *et al*., 2005; Vingada *et al*., 2010). Farmers usually check their cattle once or twice a week, otherwise leaving them unattended without livestock-guarding dogs. In winter, cattle may be assembled in stables at nightfall as shelter from severe weather conditions. Cattle comprise > 30% of the diet of wolves in Northwestern Portugal (Álvares *et al*., 2015).

### Sample collection

Hair was collected from December 2021 to November 2023, from wolf-preyed and live free-ranging cattle (Fig. 1).Wolf-preyed cattle were reported to the Portuguese Nature Conservation Authority (ICNF) for compensation. Hair samples were collected from live cattle at their stables while being handled for routine procedures such as vaccination and deworming. Cattle were handled by their owners according to routine practices, usually in a chute, and two to three veterinarians were always present (the veterinarian in charge of the clinical practice and one or two from the research team). Animals were released afterwards to move freely across the study area. Hair samples (approximately 80 mg) were collected with scissors from the forehead of cattle (between the horns or ears), as close to the skin as possible, and stored in paper envelopes at room temperature, protected from direct sunlight until analysis. Areas contaminated by blood or soil were avoided when collecting the hair sample.

Individual data was gathered from each cattle, including species, age class, sex, geographical location, date of sample collection and date of death. The date of death assigned to wolf-preyed cattle was the date the animal owner reported the wolf attack to ICNF for compensation. The animals’ age class was obtained through reports from the owner or estimated according to tooth development and classified as subadult (from birth until deciduous teeth are fully developed, usually until 12 months of age) or adult (as permanent teeth begin to be replaced) (Eubanks,  2012). Sex was established based on the external genitalia or other anatomic features such as horn morphology. Furthermore, each sample was assigned to individual farms (farm ID). Live animals were sampled at their stables, so farm ID was straightforward to attribute. Regarding preyed cattle, that information was not available due to confidentiality issues, so they were assigned a farm ID based on the sampling location. Dead and live animals were distributed across 15 farm IDs, with 1.6 ± 0.7 wolf-preyed animals/farm ID and 18 ± 14.5 live animals/farm ID (mean ± standard deviation).

### Ethical declaration

According to the Portuguese (Decreto-Lei 113/2013 of 7 August 2013) and European legislation (Directive 2010/63/EU of the European Parliament and of the Council of 22 September 2010 on the protection of animals used for scientific purposes), this study is not considered animal experimentation and does not require ethical evaluation. In the mentioned legislation, animal experimentation is defined as practices likely to cause pain, suffering, distress or lasting harm equivalent to, or higher than, that caused by the introduction of a needle in accordance with good veterinary practice. The sole procedure performed on live animals in this study was the non-invasive collection of hair samples by cutting the hair with scissors, clearly falling below the threshold of distress considered in the legislation.

### Hair cortisol extraction

The protocol used for cortisol extraction was adapted from previous research (Pereira *et al*., 2022). Hair samples were washed twice with 40 μl of distilled water/milligram of hair for 1 min followed by two 1-min washes with 40 μl of isopropanol/milligram of hair to remove surface contaminants, such as mud and vegetation. Between each washing cycle, the samples were vortexed, the supernatant discarded and the hair samples dried in a clean paper towel. After decontamination, the samples were left overnight inside the falcon tubes without caps at room temperature. In an Eppendorf PP screw cap plastic tube, the washed hair was added with two 4-mm steel beads. Hair fibres were ground into a fine powder using a Mini-Beadbeater96 (BioSpec, Bartlesville, USA) for 12 s/mg of hair at 4800 oscillations/min. Afterwards, 50 μl of methanol per mg of hair were added to each Eppendorf, and the tubes were sonicated (Izasa Scientific, Portugal) for 30 min at 50 Hz at 50°C and incubated for 18 h at 50°C. After cortisol extraction, the samples were centrifuged (Thermo Fisher Scientific, USA) for 15 min at 14 000 g at 20°C, and the supernatant was collected into a screw cap glass vial and the volume was registered. The collected supernatant was dried at room temperature under a fume hood.

### Hair cortisol quantification

A commercial competitive ELISA kit (Demeditec Cortisol free in Saliva ELISA, Germany) was used to quantify the HCC following the manufacturer’s specifications. Briefly, samples were reconstituted in 5% methanol in PBS at 17 μl solution/100 μl methanol extracted, vortexed for 30 s and 50 μl were added to each well, as well as the standard solutions and controls. Cortisol-horseradish peroxidase conjugate was added to each well (50 μl), mixed for 10 s, incubated for 60 min at room temperature and washed four times with 300 μl wash solution in a BioTek ELx50 microplate strip washer (Agilent, Santa Clara, USA). Two hundred μl of substrate solution was added to each well, incubated in the dark at room temperature for 30 min, the reaction stopped with 50 μl of stop solution and the optical density (OD) read at 450 nm (iMark Reader, Bio-Rad, Hercules, USA).

The software GraphPad Prism (GraphPad Software version 8.0.1, La Jolla, California, USA) was used to determine the four-parameter logistic curve using the OD and log-transformed cortisol concentrations of the standards provided with the kit (R^2^adjusted 0.998–0.999). The cortisol concentration of the reconstituted samples was estimated from the standard curve and converted to cortisol concentration as picograms (pg) of cortisol/mg of hair.

The analytical performance of this protocol has been previously established in wolf (Pereira *et al*., 2022) and cattle hair (Nedić *et al*., 2017). Intra- and inter-assay coefficient of variation ranged between 7.49 and 7.68% and 7.90–8.93% for low and high cortisol concentrations, respectively (Nedić *et al*., 2017). According to the manufacturer, the cross-reactivity of the test is < 0.5% for the corticosteroids tested, except for 11-deoxycortisol (10.2%), corticosterone (5.2%) and prednisolone (63.4%).

### Covariates

The variables analysed were age, sex, status (i.e. whether it was alive or preyed), time of exposure (number of days the hair was exposed to weather conditions between death and sample collection) and food availability. These variables have been chosen to control for intrinsic biological factors (sex and age), post-mortem conditions (weather exposure) and ecological context (food availability), all of which could influence HCC. Age and sexual dimorphism influence the HPA axis activity, where baseline cortisol levels vary across developmental and life stage processes, as well as between females and males, leading to differences in hair cortisol deposition (Heimbürge ***et al***., 2020). Previous studies have indicated that exposure to sunlight and UV radiation can reduce HCC (Wester ***et al***., 2016). To control for this effect, the time of exposure to environmental conditions was calculated as the number of days between the date of death and sample collection date. In live animals, this variable assumed the value zero. Food availability, which influences stress hormone levels (e.g. Bryan ***et al***., 2014), was determined through the mean normalised difference vegetation index (NDVI) of the 80 days before sample collection, reflecting the trophic resources available during the summer, autumn and winter months (between August and February). The 80-day interval was selected based on the quiescent phase of the hair cycle growth (Turner and Schleger, 1970), and after consideration of different intervals (1-, 2- or 3-month intervals) and their influence on HCC. The NDVI was measured in a 2-km buffer centred on the GPS coordinates where dead animals were found or the approximate centroid of the pastures of live cattle. Live cattle were sampled at their stables, which are usually removed from pastures. In these cases, the approximate location of the pastures was established by interviews with cattle owners.

### Statistical analysis

All analyses were carried out using R version 4.2.3 (R Core Team, 2023), through the interface RStudio 2023.12.1 + 402, and linear mixed models with Gaussian distribution and identity link were implemented using the package ‘lme4’ (Bates *et al*., 2015). Reference classes for the categorical variables were ‘female’, ‘adult’ and ‘preyed’. Continuous variables (NDVI and time of exposure) were standardised to their *z*-score. The model assumptions of normality of residuals and homoscedasticity were assessed through QQ and residuals-predicted values plots. One influential outlier observation driving the model results was identified through the QQ plots and removed from the analysis (Table S1).

All models included a random effect of farm ID to accommodate potential variation of HCC across farms and lack of independence between results of samples from the same farm. Food availability was included in the models as the quadratic expression of the mean of the NDVI from the 80 days before sample collection (NDVI^2^). The initial model included the independent variables age, sex and status (categorical), time of exposure and food availability (continuous) and the interaction between age and food availability. An interaction between age and status was considered in a first analysis, although it was a non-significant variable for HCC and reduced strength to the final model. Models were ranked using Akaike’s Information Criterion (AIC) (Burnham and Anderson, 2002) and a conditional average of the ones presenting ΔAIC<2 was implemented using the package ‘MumIn’ (Bartoń, 2024) in R.

## Results

A total of 64 samples were included in the analysis, of which 46 were from live cattle (7 males, 39 females; 37 adults, 9 subadults) and 19 were wolf-preyed cattle (7 males, 12 females; 11 adults, 8 subadults) (Table S1). Extensive farming practises in northern Portugal often correspond to a higher ratio of cows to bulls (Araujo *et al*., 2014), which is reflected in the number of samples from females and male cattle, both live and wolf-preyed. The HCC ranged from 1.2 to 16.7 pg/mg, with a mean ± standard deviation of 7.3 ± 4.1 pg/mg. The initial model included the fixed effects of age, sex, status, time, food availability (quadratic effect), the interaction between age and food and the random effect of farm ID. The conditional coefficient of determination of the initial model was R^2^_c_ = 0.806. The three selected models with a ΔAIC<2 (Table 1) were averaged, with a variable weight of 1.00 for age, status, food and the interaction of age and food, 0.33 for sex and 0.19 for time of exposure. The conditional coefficient of determination of the averaged model was R^2^_c_ = 0.659.

**Table 1:** Comparison of linear mixed-effect models developed for the response variable of HCC of cattle

Model	Model description	AIC	ΔAIC	Weight
1	**HCC ~ (1|farm ID) + status + age + food + age × food**	**319.9**	**0.00**	**0.387**
2	HCC ~ (1|farm ID) + status + age + food + sex + age × food	320.6	0.70	0.273
3	HCC ~ (1|farm ID) + status + age + food + time + age × food	321.8	1.84	0.154
Full model	HCC ~ (1|farm ID) + age + status + food + sex + time + age × food	322.5	2.54	0.109
Null model	HCC ~ (1|farm ID)	340.6	20.69	0.00

Models with ΔAIC < 2, full and null models are presented and include farm ID as a random effect. Each model presents the corresponding AIC, ΔAIC compared to the best model represented in bold, and the weight of the model. Model variables include: status (alive or preyed), age (subadult or adult), sex (female or male), food (food availability, obtained from normalised difference vegetation index), time (number of days of exposure to environmental conditions) and ‘age × food’ interaction.

**Figure 2:**
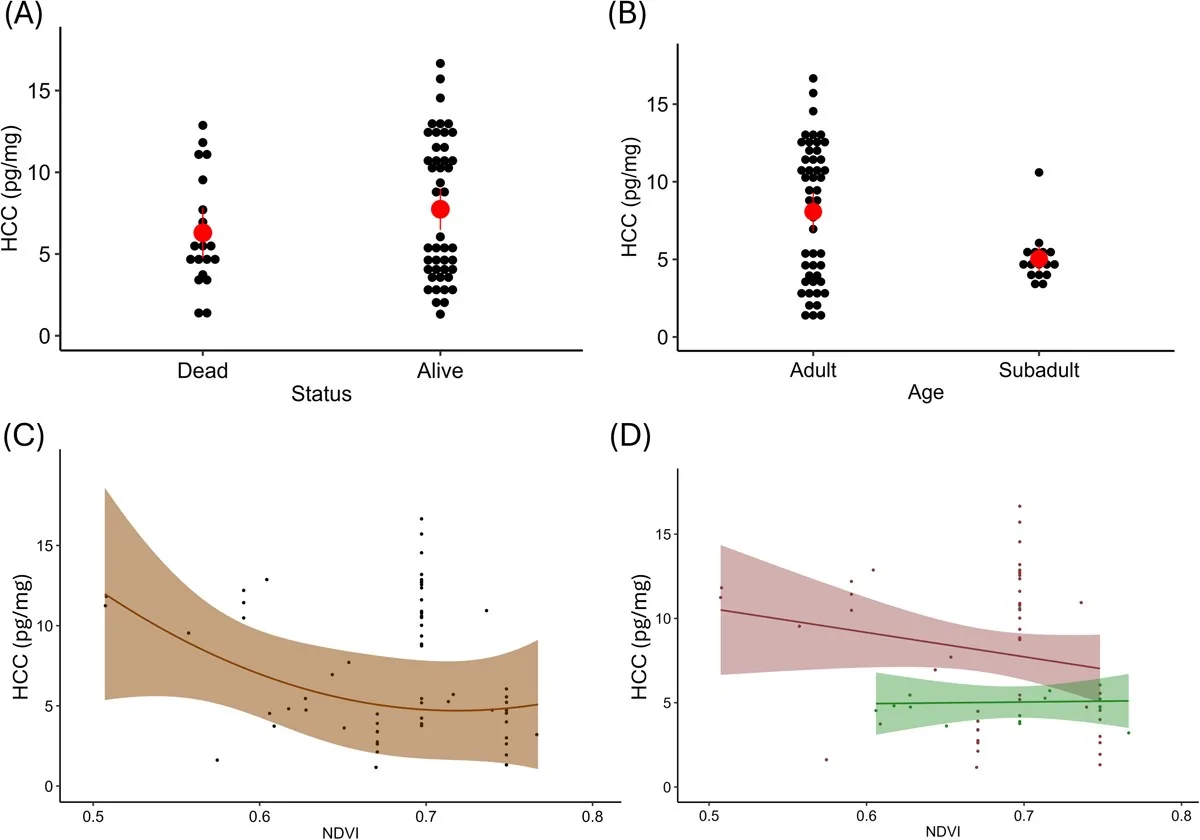
Significant effects of the variables on HCC. Mean and confidence interval of HCC according to the (A) status (alive or preyed by wolves), and (B) age class. Linear relationship and 95% confidence intervals (shaded areas) of (C) food availability as the quadratic effect of the normalised difference vegetation index on the 80 days before being preyed upon, and (D) the interaction between food availability and age class (adults in red and subadults in green).

Live cattle exhibited significantly (*P* = 0.009) higher HCC (7.7 ± 4.3 pg/mg) than wolf-preyed cattle (6.3 ± 3.5 pg/mg) (Fig. 2A and Table 2). The HCC was significantly correlated with age (*P* < 0.001), with subadults presenting lower HCC than adults (8.1 ± 4.4 pg/mg vs 5.0 ± 1.7 pg/mg) (Fig. 2B and Table 2). Food availability showed a significant positive quadratic effect on HCC (*P* = 0.041) (Fig. 2C and Table 2). The interaction between age and food availability was significantly correlated with HCC (*P* = 0.004), with a negative relationship in adults but not in subadults (Fig. 2D and Table 2). Sex had a non-significant effect on HCC (*P* = 0.992). The time variable measuring exposure to weather conditions (mean of 7.5 ± 6.6 days for dead animals) was not statistically significant to HCC (Table 2). Time exposure presented a negative relationship to HCC, and there was no visible evidence of damage to hair samples with longer time exposure.

**Table 2:** Summary of the averaged model

Variable	Estimate (β)	Standard error (SE)	*P*-value	Confidence interval (95%)
Intercept	4.404	1.468	0.003	[1.464–7.343]
Status (alive)	5.281	2.003	**0.009**	**[1.270–9.294]**
Age (subadult)	−5.019	1.230	**<0.001**	**[−7.483 to − 2.556]**
Sex (male)	−0.007	0.604	0.992	[−2.111 to 2.072]
Food	0.812	0.390	**0.041**	**[0.031–1.592]**
Time	−0.081	0.586	0.893	[−1.254 to 1.093]
Age × food	3.512	1.186	**0.004**	**[1.137–5.887]**

The farm ID was included as a random effect. The values with a significant correlation (*P* < 0.05) are expressed in bold. Model variables include: status (alive or preyed), age (subadult or adult), sex (female or male), food (food availability, obtained from normalised difference vegetation index), time (number of days of exposure to environmental conditions) and ‘age x food’ interaction.

## Discussion

This study aimed to assess whether HCC was a biomarker of wolf prey selection on free-range cattle, correcting for the effect of other potential determinants of HCC. Our results show a significant difference in HCC between alive and wolf-preyed cattle, supporting HCC as one possible biomarker of wolf predation risk. However, contrary to the initial hypothesis, wolf-preyed cattle presented lower HCC.

Shave *et al*. (2019) demonstrated that wolf-preyed bison exhibited higher HCC and lower body condition, which was associated with a higher vulnerability to predation, opposite to what we found in free-range cattle. Although we did not assess body condition, assuming the relationship between HCC and body condition was the same as for bison, our results suggest that wolves select prey with higher body condition. This opposite result, compared to bison prey, could be related to the differential effort required to take down large wild prey, such as bison [average weights 360–900 kg (Licht, 2016)], compared to small-sized free-range cattle. According to this explanation, preying on bison would require wolves to select individuals with lower body condition, with higher HCC. In Northwestern Portugal, free-range cattle are mostly small-sized [~250 kg (Brito *et al*., 2005)]; as such, the effort required to prey on individuals with high or low body condition may not be much different. Wolves may be targeting healthier or higher body-conditioned livestock individuals, since, in contrast to wild ungulates, livestock presents lower defensive abilities (e.g. less agility), possibly representing a lower risk of injuries and fewer unsuccessful predation attempts. However, empirical evidence in this regard remains limited.

Another possible explanation for our results is related to anti-predatory behaviour, which can vary among individuals. Some individual cattle may be less vigilant, with reduced anti-predatory behaviours and, consequently, be at higher risk of predation. In turn, other individuals can be more vigilant (Kluever *et al*., 2008; Laporte *et al*., 2010) and may exhibit more anti-predatory behaviours that increase fitness (i.e. survival) and have higher HCC due to chronic stress. Studies have indicated that herding animals tend to group together for the benefits of collective vigilance and defence, and risk dilution (Hebblewhite and Pletscher, 2002; Childress and Lung, 2003). Though, other studies report opposite results, with smaller herd sizes when predators are present (Creel and Winnie, 2005) and an increase of vigilance in large groups due to visual obstruction (Kluever *et al*., 2008). Additionally, other potential determinants of HCC, not addressed in this study, could be used as clues by wolves to select individual cattle. These indicators could include the animals’ body condition (Shave *et al*., 2019) or parasitic infestation, particularly with ectoparasites (Vitela-Mendoza *et al*., 2016), as gastrointestinal infections were shown not to influence HCC in reindeer (*Rangifer tarandus*) (Carlsson *et al*., 2016). Further studies considering the frequency of wolf attacks, cattle body condition and behaviour, parasitism, and herd size are needed to understand how cattle fitness and anti-predatory behaviours are related to HCC.

Our results support that HCC is significantly higher in adults than subadults. Previous studies have documented differences in HCC according to age (González-de-la-Vara *et al*., 2011; Nejad *et al*., 2019; Heimbürge *et al*., 2020), including for livestock under extensive production systems (Nedić *et al*., 2017). Contrary to our results, Heimbürge *et al*. (2020) reported higher HCC in newborn calves (>50 pg/mg) than cattle with 6 months, 18 months, or adults (<10 pg/mg), attributing it to the secretion of foetal glucocorticoid at the end of gestation (Flint *et al*., 1979; González-de-la-Vara *et al*., 2011). On the other hand, similarly to our results, Nejad *et al*. (2019) reported significantly higher HCC in adult cows (8–10 pg/mg) than heifers (10–12 months; 6 pg/mg). The differences in HCC between adults and juveniles, other than newborns, may be attributed, e.g. to longer exposure to environmental stressors, like extreme temperatures (Ripamonti *et al*., 2025), clinical diseases or the reproductive stage cycle of adult individuals, such as pregnancy (Burnett *et al*., 2015; Edwards and Boonstra, 2018) or lactation (Nejad *et al*., 2019). Hence, adults are exposed to a broad range of long-lasting stressors—including physiological stressors—contributing to chronic stress and elevated HCC. Whereas young animals typically face acute stressors, such as weaning, which has a lower cumulative exposure.

Our results show that HCC tends to decrease in adult cattle with increasing food availability up to an NDVI of ~ 0.7, then stabilising at low levels. Food availability has been shown to modify HCC and behavioural responses among livestock both in intensive and pasture production systems (Herskin *et al*., 2003; Poudel *et al*., 2022). Although the direct impact of food deprivation on cattle’s HCC is not explicitly detailed in previous studies, the associated physiological changes suggest potential implications. In reduced food availability scenarios, animals increase competitive behaviours towards scarce resources and decrease their health and welfare conditions, suggesting a potential effect of nutritional stress (Hogan and Phillips, 2016). HCC has been positively correlated with a decrease in body condition in livestock (Endo *et al*., 2019), pigs (Trevisan *et al*., 2017) or bears (Bryan *et al*., 2014; Cattet *et al*., 2014), associated with the mobilisation of energy storage. In addition, food shortages can lead to increased foraging behaviours (Norberg, 2021) that could induce animals to move towards unfamiliar areas, increase the probability of encountering predators, and, therefore, increase HCC.

Furthermore, in our study, the effect of food availability was modulated by age, with HCC in subadults being independent of NDVI. These results support that lower food availability corresponds to significantly higher HCC in adults, while subadults were not affected. Juvenile cattle depend on a mixture of milk provided by their progenitor and vegetation available throughout the weaning process until 10 months of age. Adults feed exclusively on available vegetation (Reinhardt and Reinhardt, 1981), as supplemental feeding is rarely employed in our study area.

While this study provides valuable insights, some limitations hinder further conclusions. We were only able to collect samples from 19 wolf-killed cattle, which may limit the generalisation of the results. In addition, samples from live animals should encompass all the territory where wolf-attack samples were collected, even though live animals graze freely across the study area for most of the year. This limitation may influence the results, as spatial variables derived from sample collection locations may vary across the study area. Furthermore, the samples collected in our study were unevenly distributed across age classes (Fig. S1), which may contribute to the apparent difference in HCC between predation statuses. Ideally, such distribution should be even to further evaluate the possible implications associated with age and the secretion and incorporation of HCC. However, due to budget constraints, we were unable to collect and analyse further samples, either from live or dead animals, which could have otherwise provided additional robustness and valuable insights into the potential determinants for HCC. Furthermore, comparing different matrices that incorporate cortisol throughout time, such as hair or nails (Gormally and Romero, 2020) or the inclusion of climatic variables, such as temperature or precipitation, previous to death, may be a further consideration for future research. These metrics may provide additional information on the most adequate matrix to evaluate the degree of chronic stress to which animals have been exposed and the factors influencing HCC.

This study reframes the predator–prey dynamic, highlighting a lower HCC in wolf-preyed cattle. Our results emphasise the value of HCC in identifying cattle susceptible to predation, allowing targeted management strategies towards animals with lower HCC. It also raises the possibility that, while cattle frequently exposed to predation may exhibit higher HCC, they may also develop more effective anti-predatory behaviours. Even though conflicts arise when livestock depredations occur, coexistence is of utmost importance, since wolves play a critical part in ecosystems as top predators and regulators of ecological health (Licht *et al*., 2010; Frey *et al.*, 2022), hence their maintenance in the ecosystem, even though with livestock casualties, is of vital importance. Although human–wolf conflicts often result in lethal control measures against wolves, non-lethal strategies should be valued in reducing conflicts and promoting large carnivore conservation (van Eeden *et al.*, 2018). Thus, targeted livestock management based on physiological and behavioural indicators, such as HCC, can help mitigate these losses. Livestock management strategies could include the relocation and increased supervision of vulnerable individuals (i.e. with lower HCC). Furthermore, the inclusion of vulnerable individuals in more cohesive or larger groups could amplify natural protective behaviours (Pays *et al.*, 2007; Tobin *et al.*, 2021) and enhance predator detection and avoidance. By reducing predation through such proactive measures, livestock owners would be less likely to advocate for wolf removal, supporting a more sustainable coexistence.

## Conclusions

This study identified a link between HCC in free-range cattle and predation, suggesting that wolves tend to prey on animals with lower HCC. Furthermore, it illustrates the age class-related relationship between food availability and HCC, which was negative for adult cattle, while no relationship was found for subadults. Multiple questions remain unanswered, and further research is required to better understand the HCC-related drivers of wolf prey selection. Knowledge of this relationship, as well as the integration of additional information on prey, including body condition, parasitic status, anti-predatory behaviour or herd size, could translate into improved management of predation by wolves on free-range cattle. Such insights would, thus, contribute to more effective conservation strategies, and promote human–wildlife coexistence in human-dominated landscapes.

## Supplementary Material

Web_Material_coag002

## Data Availability

All data supporting the findings of the present study are available as Supplementary Material.
