# Correction to: Could hair cortisol in free-ranging cattle be a proxy of wolf predation patterns? 

**DOI:** 10.1093/conphys/coag065

**Published:** 2026-08-31

This is a correction to: Marta Rafael, Eliana Fonseca, Nuno Santos, Mónia Nakamura, Could hair cortisol in free-ranging cattle be a proxy of wolf predation patterns?, *Conservation Physiology*, Volume 14, Issue 1, 2026, coag002, https://doi.org/10.1093/conphys/coag002.

In April 2026, the journal was made aware of some omissions from the Materials and Methods and Ethical Declaration sections of this article. The journal has prepared this correction notice in collaboration with the authors, and the article has been corrected to include further details of the sample collection procedures and of the ethical legislations followed. 

 In the originally published version of this manuscript, details of the sample collection methodology were omitted from the ``sample collection'' section. This section originally read: 

 ``Hair was collected from December 2021 to November 2023, from wolf-preyed and live free-ranging cattle (Fig. 1). Wolf-preyed cattle were reported to the Portuguese Nature Conservation Authority (ICNF) for compensation. Hair samples were collected from live cattle at their stables while being handled for routine procedures such as vaccination and deworming. They were released afterwards to move freely across the study area. Hair samples were collected with scissors from the forehead of cattle (between the horns or ears), as close to the skin as possible, and stored in paper envelopes at room temperature, protected from direct sunlight until analysis. Areas contaminated by blood or soil were avoided when collecting the hair sample.''

This paragraph has now been corrected to read:

``Hair was collected from December 2021 to November 2023, from wolf-preyed and live free-ranging cattle (Fig. 1). Wolf-preyed cattle were reported to the Portuguese Nature Conservation Authority (ICNF) for compensation. Hair samples were collected from live cattle at their stables while being handled for routine procedures such as vaccination and deworming. Cattle were handled by their owners according to routine practices, usually in a chute, and two to three veterinarians were always present (the veterinarian in charge of the clinical practice and one or two from the research team). Animals were released afterwards to move freely across the study area. Hair samples (approximately 80 mg) were collected with scissors from the forehead of cattle (between the horns or ears), as close to the skin as possible, and stored in paper envelopes at room temperature, protected from direct sunlight until analysis. Areas contaminated by blood or soil were avoided when collecting the hair sample.''

In addition, details of the specific legislations followed were omitted from the Ethical declaration section of the article. This section originally read:

``No cattle were physically contained for the sole purpose of this study, and samples were collected using non-invasive procedures. According to the Portuguese and European legislation, this study is not considered animal experimentation and does not require ethical evaluation''.

This section has been corrected to read:

``According to the Portuguese (Decreto-Lei 113/2013 of 7 August 2013) and European legislation (Directive 2010/63/EU of the European Parliament and of the Council of 22 September 2010 on the protection of animals used for scientific purposes), this study is not considered animal experimentation and does not require ethical evaluation. In the mentioned legislation, animal experimentation is defined as practices likely to cause pain, suffering, distress or lasting harm equivalent to, or higher than, that caused by the introduction of a needle in accordance with good veterinary practice. The sole procedure performed on live animals in this study was the non-invasive collection of hair samples by cutting the hair with scissors, clearly falling below the threshold of distress considered in the legislation''.

The version of record has been corrected directly.

