# Supplementary material for: Could hair cortisol in free-ranging cattle be a proxy of wolf predation patterns?
Source: Conserv Physiol. 2026 Jan 28;14(1):coag002. doi: 10.1093/conphys/coag002 (PMC12851633; doi:10.1093/conphys/coag002)
Supplement: Web_Material_coag002 [file web_material_coag002.zip › Supplementary_material .pdf]

Supplementary Material

Manuscript title: Cortisol in the hair of free-ranging cattle as a possible proxy of wolf predation patterns

Table S1. Hair samples collected and analysed from wolf preyed (n = 19) and alive (n = 46) free-range cattle in Northwest Portugal.

Sample ID	Date of sample collection	Farm ID	NDVI	Date of Death	Sex	Age	Status	Time exposed to environmental conditions (days) [a]	Cortisol concentration (pg/mg)
**7**	12/01/2022	4	0.713	10/01/2022	Female	Subadult	Preyed	2	5.3
**8**	02/12/2021	7	0.736	30/11/2021	Male	Adult	Preyed	2	10.9
**9**	18/12/2021	8	0.644	02/12/2021	Female	Adult	Preyed	16	6.9
**10**	18/12/2021	10	0.604	06/12/2021	Male	Adult	Preyed	12	12.9
**12**	13/01/2022	7	0.716	29/12/2021	Female	Subadult	Preyed	15	5.7
**13**	13/01/2022	11	0.740	30/12/2021	Female	Adult	Preyed	14	4.7
**15**	13/01/2022	12	0.766	22/12/2021	Female	Subadult	Preyed	22	3.2
**16**	14/01/2022	15	0.575	14/01/2022	Female	Adult	Preyed	0	1.6
**17**	31/01/2022	1	0.670	19/01/2022	Female	Adult	Preyed	12	1.2
**18**	01/02/2022	14	0.609	24/01/2022	Female	Adult	Preyed	8	3.7
**19**	19/02/2022	9	0.508	14/02/2022	Female	Adult	Preyed	5	11.8
**20**	19/02/2022	9	0.507	14/02/2022	Female	Adult	Preyed	5	11.2
**21**	19/02/2022	6	0.617	01/02/2022	Male	Subadult	Preyed	18	4.8
**23**	19/03/2022	8	0.606	15/03/2022	Male	Subadult	Preyed	4	4.5

Sample ID	Date of sample collection	Farm ID	NDVI	Date of Death	Sex	Age	Status	Time exposed to environmental conditions (days) [a]	Cortisol concentration (pg/mg)
**24**	29/03/2022	5	0.628	15/03/2022	Male	Subadult	Preyed	14	5.5
**25**	29/03/2022	5	0.628	25/03/2022	Male	Subadult	Preyed	4	4.7
**26**	28/04/2022	4	0.654	09/04/2022	Female	Adult	Preyed	19	7.7
**27**	28/04/2022	4	0.651	28/04/2022	Male	Subadult	Preyed	0	3.6
**28**	28/04/2022	14	0.557	27/04/2022	Female	Adult	Preyed	1	9.5
**30**	28/04/2022	2	0.697	NA	Male	Subadult	Alive	0	3.8
**31**	28/04/2022	2	0.697	NA	Male	Subadult	Alive	0	4.2
**32**	28/04/2022	2	0.697	NA	Male	Subadult	Alive	0	3.9
**33**	28/04/2022	1	0.590	NA	Female	Adult	Alive	0	12.2
**34**	28/04/2022	1	0.590	NA	Female	Adult	Alive	0	11.4
**35**	28/04/2022	1	0.590	NA	Female	Adult	Alive	0	10.5
**36**	28/04/2022	2	0.697	NA	Male	Adult	Alive	0	14.5
**37**	28/04/2022	2	0.697	NA	Female	Adult	Alive	0	8.7
**38**	28/04/2022	2	0.697	NA	Female	Subadult	Alive	0	10.6
**39**	28/04/2022	2	0.697	NA	Female	Adult	Alive	0	11.6
**40**	28/04/2022	2	0.697	NA	Female	Adult	Alive	0	12.7
**41**	28/04/2022	2	0.697	NA	Female	Adult	Alive	0	10.5
**42**	28/04/2022	2	0.697	NA	Female	Adult	Alive	0	10.8

Sample ID	Date of sample collection	Farm ID	NDVI	Date of Death	Sex	Age	Status	Time exposed to environmental conditions (days) [a]	Cortisol concentration (pg/mg)
**43**	28/04/2022	2	0.697	NA	Female	Adult	Alive	0	10.9
**44**	28/04/2022	2	0.697	NA	Female	Adult	Alive	0	16.7
**45**	28/04/2022	2	0.697	NA	Female	Adult	Alive	0	10.5
**46**	28/04/2022	2	0.697	NA	Female	Adult	Alive	0	12.4
**47**	28/04/2022	2	0.697	NA	Female	Adult	Alive	0	12.7
**48**	28/04/2022	2	0.697	NA	Female	Adult	Alive	0	13.2
**49**	28/04/2022	2	0.697	NA	Female	Adult	Alive	0	5.5
**54**	28/04/2022	2	0.697	NA	Female	Adult	Alive	0	9.4
**57**	28/04/2022	2	0.697	NA	Female	Adult	Alive	0	12.6
**58**	28/04/2022	2	0.697	NA	Female	Adult	Alive	0	5.2
**59**	28/04/2022	2	0.697	NA	Female	Adult	Alive	0	10.0
**60**	28/04/2022	2	0.697	NA	Female	Adult	Alive	0	12.9
**61**	28/04/2022	2	0.697	NA	Female	Adult	Alive	0	8.9
**64**	28/04/2022	2	0.697	NA	Female	Adult	Alive	0	15.7
**66**	16/12/2022	3	0.671	NA	Female	Adult	Alive	0	3.4
**67**	16/12/2022	3	0.671	NA	Female	Adult	Alive	0	4.5
**68**	16/12/2022	3	0.671	NA	Male	Subadult	Alive	0	9.3
**69**	16/12/2022	3	0.671	NA	Female	Adult	Alive	0	2.8

Sample ID	Date of sample collection	Farm ID	NDVI	Date of Death	Sex	Age	Status	Time exposed to environmental conditions (days) [a]	Cortisol concentration (pg/mg)
**70**	16/12/2022	3	0.671	NA	Female	Adult	Alive	0	3.4
**71**	16/12/2022	3	0.671	NA	Female	Adult	Alive	0	3.9
**72**	16/12/2022	3	0.671	NA	Female	Adult	Alive	0	2.1
**73**	16/12/2022	3	0.671	NA	Female	Adult	Alive	0	2.6
**74**	01/11/2023	3	0.748	NA	Male	Subadult	Alive	0	5.2
**75**	01/11/2023	3	0.748	NA	Male	Subadult	Alive	0	4.7
**76**	01/11/2023	3	0.748	NA	Female	Subadult	Alive	0	6.1
**77**	01/11/2023	3	0.748	NA	Female	Subadult	Alive	0	4.8
**78**	01/11/2023	3	0.748	NA	Female	Adult	Alive	0	3.0
**79**	01/11/2023	3	0.748	NA	Female	Adult	Alive	0	5.6
**80**	01/11/2023	3	0.748	NA	Female	Adult	Alive	0	4.5
**81**	01/11/2023	3	0.748	NA	Female	Adult	Alive	0	1.3
**82**	01/11/2023	3	0.748	NA	Female	Adult	Alive	0	1.9
**83**	01/11/2023	3	0.748	NA	Female	Adult	Alive	0	4.0
**84**	01/11/2023	3	0.748	NA	Female	Adult	Alive	0	2.6

[a] Calculated as the difference between the date of sample collection and date of death.

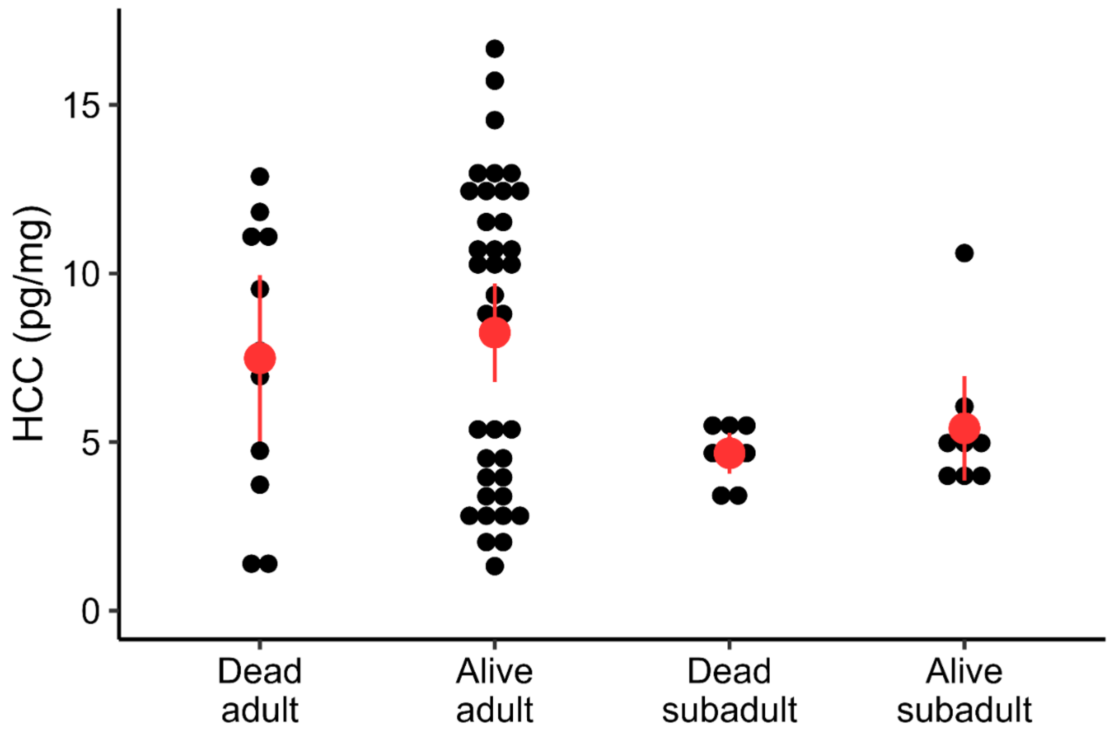

Figure S1. Hair cortisol concentration (HCC) across age-status classes. Black points represent individual observed HCC values. Mean and 95% confidence intervals of HCC for each age-status class (adult or subadult; alive or dead) are represented in red.
