# Supplementary figures and images for: Could hair cortisol in free-ranging cattle be a proxy of wolf predation patterns?
Source: Conserv Physiol. 2026 Jan 28;14(1):coag002. doi: 10.1093/conphys/coag002 (PMC12851633; doi:10.1093/conphys/coag002)

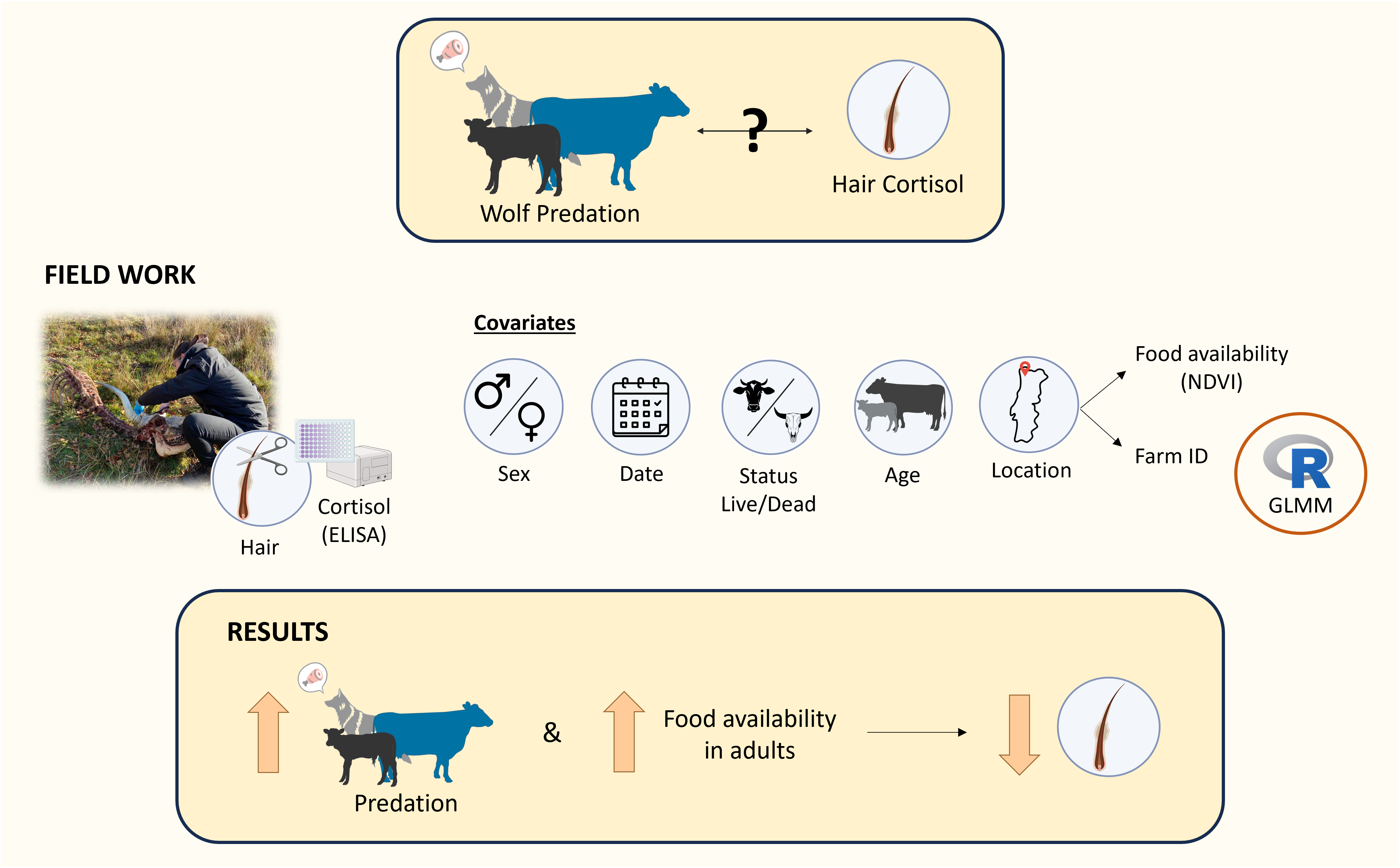

Supplement: Web_Material_coag002 [file web_material_coag002.zip › Graphical abstract.tif]
